# The ICD-11 is coming to town! Educational needs, paradigm shifts and innovations in mental health care practice

**DOI:** 10.1192/j.eurpsy.2021.2254

**Published:** 2021-11-24

**Authors:** Andrea Fiorillo, Peter Falkai

**Affiliations:** 1 Department of Psychiatry, University of Campania “L. Vanvitelli”, Naples, Italy; 2 Department of Psychiatry and Psychotherapy, Ludwig-Maximilians-University Munich, Munich, Germany

**Keywords:** Classification systems, education, mental disorder, mental health care

Classification systems are essential tools in routine clinical work for providing reliable and clinically useful diagnoses, supporting clinicians and health care workers in identifying patients with higher health needs, and guiding the implementation of the best available care according to the diagnosis [1][2]. In psychiatric practice, classification systems are useful to improve communication among mental health professionals and researchers and to establish widely agreed descriptions of mental disorders, and should not be considered textbooks of psychopathology. Moreover, classification systems offer a framework for education on the most common clinical features of mental disorders through the organization of disorders into discrete diagnostic categories [3][4]. A clear and straightforward classification system can help clinicians to communicate diagnosis to patients and their family members, and to reduce stigma attached to mental disorders. Finally, diagnostic systems facilitate the identification and management of mental disorders in clinical settings and have a significant predictive power [5]. It is understood, however, that they should be complemented by a more detailed clinical characterization of each individual patient [6] [7].

The two most widely used classification systems in mental health are the Diagnostic and Statistical Manual of Mental Disorders (DSM), issued by the American Psychiatric Association (APA), and the International Classification of Diseases (ICD), produced by the World Health Organization (WHO). The latest version of the DSM was published in 2013, whereas the 11th revision of the ICD by the WHO has been completed in 2018 and approved by the WHO General Assembly in 2019 [8]. The official reporting of health statistics by Member States to the WHO using the ICD-11 will begin on January 1, 2022 [9].

The development of the ICD-11 chapter on mental, behavioral, and neurodevelopmental disorders represents the first major revision of the world’s foremost classification of mental disorders, which took nearly 30 years to be completed. In fact, the revision of the ICD-11 represents the biggest global, multidisciplinary, and participative process of revision of a classification system for mental disorders which has ever been implemented [9]. Its development has involved the collaboration among several stakeholders, including some of the most eminent scientists in the field, international scientific associations, and organizations of users and carers [10] [11]. Furthermore, the WHO Global Clinical Practice Network, an international network including more than 16,000 clinicians from 159 countries, has been involved in the field trials of the diagnostic system [9][12]. The ICD-11 is currently being translated in several languages in order to be used in routine clinical practice in different parts of the world [13].

Compared with the previous versions, the ICD-11 presents several innovative features, such as the lifespan approach to mental disorders, the inclusion of a dimensional component within a system which remains mainly categorically based, and the inclusion of culture-related information [14][15][16][17][18][19][20][21]. The ICD-11 Clinical Descriptions and Diagnostic Guidelines (CDDG) have introduced substantial changes in order to improve the clinical utility and global applicability of diagnoses of mental disorders in routine clinical care. In particular, the guidelines provide the essential features of each disorder, including symptoms or characteristics that a clinician could reasonably expect to find in all patients affected by the same disorder [9]. Furthermore, no cutoffs and precise diagnostic requirements are listed, unless these are empirically established across countries and cultures, in order to provide a diagnostic tool which is as close as possible to real-world clinical practice. A flexible approach has been endorsed, so that the manual can be adapted by clinicians to their clinical routine care [9][22]. The feasibility of CDDG has been confirmed by the ICD-11 field trials, which were well received by clinicians and documented a high inter-rater reliability and accuracy of diagnostic categories[23][24][25].

In order to adapt the classification system to the local countries’ laws, policies, health systems, and infrastructures, several multilevel actions have been subsequently implemented. Moreover, since education of health care professionals represents one of the most essential steps for the implementation and the dissemination of the new classification system in routine care, the WHO International Advisory Group led by G. M. Reed has organized training courses for professionals on the use of the ICD-11 chapter on mental, behavioral, and neurodevelopmental disorders and the relevant CDDG. Educational activities have been provided through virtual interactive formats, including an online course organized in collaboration with the European Psychiatric Association (EPA) from April 9 to 30, 2021, with the participation of several clinicians from European and non-European countries. This course was attended by 120 psychiatrists, selected from almost 500 applicants, representing 78 different countries from all over the world (e.g., from Austria, Italy, and Germany to the United States, Japan, and Thailand). During the online course, the key principles of the WHO’s ICD-11 and CDDG have been presented and discussed, with the involvement of world leaders in the field who participated in the development of CDDG and the active participation of trainees through the application of the new guidelines to clinical cases and discussion of diagnostic dilemmas. Previous training initiatives had been conducted during the 18th and 19th World Congresses of Psychiatry [26][27][28][29].

The adoption of this new classification system will represent a major change in psychiatric clinical practice worldwide. There is the need to promote educational activities in order to improve the dissemination of this innovative classification approach and to contribute to the continuous education of mental health care professionals. The EPA is committed to do so through its official channels, such as Scientific Sections, National Psychiatric Associations, and the Committees on Education and on Publications.

## References

[1] Reed GM, Roberts MC, Keeley J, Hooppell C, Matsumoto C, Sharan P, et al. Mental health professionals’ natural taxonomies of mental disorders: implications for the clinical utility of the ICD-11 and the DSM-5. J Clin Psychol. 2013;69:1191–212. 24122386 10.1002/jclp.22031

[2] Keeley JW, Reed GM, Roberts MC, Evans SC, Medina-Mora ME, Robles, et al. Developing a science of clinical utility in diagnostic classification systems field study strategies for ICD-11 mental and behavioral disorders. Am Psychol. 2016;71:3–16. 26766762 10.1037/a0039972

[3] Stein DJ, Lund C, Nesse RM. Classification systems in psychiatry: diagnosis and global mental health in the era of DSM-5 and ICD-11. Curr Opin Psychiatry. 2013;26:493–7. 23867662 10.1097/YCO.0b013e3283642dfd PMC4270276

[4] Stein DJ, Reed GM. Global mental health and psychiatric nosology: DSM-5, ICD-11, and RDoC. Braz J Psychiatry. 2019;41:3–4. 10.1590/1516-4446-2018-4101 PMC6781713 30672968

[5] First MB. Paradigm shifts and the development of the diagnostic and statistical manual of mental disorders: past experiences and future aspirations. Can J Psychiatr. 2010;55:692–700. 10.1177/070674371005501102 21070696

[6] Maj M, Stein DJ, Parker G, Zimmerman M, Fava GA, De Hert M, et al. The clinical characterization of the adult patient with depression aimed at personalization of management. World Psychiatry. 2020;19:269–93. 32931110 10.1002/wps.20771 PMC7491646

[7] van Os J, Guloksuz S, Vijn TW, Hafkenscheid A, Delespaul P. The evidence-based group-level symptom-reduction model as the organizing principle for mental health care: time for change? World Psychiatry. 2019;18:88–96. 30600612 10.1002/wps.20609 PMC6313681

[8] Pocai B. The ICD-11 has been adopted by the world health assembly. World Psychiatry. 2019;18:371–2. 31496092 10.1002/wps.20689 PMC6732675

[9] Reed GM, First MB, Kogan CS, Hyman SE, Gureje O, Gaebel W, et al. Innovations and changes in the ICD-11 classification of mental, behavioural and neurodevelopmental disorders. World Psychiatry. 2019;18:3–19. 30600616 10.1002/wps.20611 PMC6313247

[10] Fuss J, Lemay K, Stein DJ, Briken P, Jakob R, Reed GM, et al. Public stakeholders’ comments on ICD-11 chapters related to mental and sexual health. World Psychiatry. 2019;18:233–5. 31059633 10.1002/wps.20635 PMC6502458

[11] Priebe S, Miglietta E. Assessment and determinants of patient satisfaction with mental health care. World Psychiatry. 2019;18:30–1. 30600613 10.1002/wps.20586 PMC6313237

[12] Reed GM, Rebello TJ, Pike KM, Medina-Mora ME, Gureje O, Zhao M, et al. WHO’s global clinical practice network for mental health. Lancet Psychiatry. 2015;2:379–80. 26360271 10.1016/S2215-0366(15)00183-2

[13] ICD-11. Implementation or transition guide. Geneva: World Health Organization; 2019.

[14] Gureje O, Lewis-Fernandez R, Hall BJ, Reed GM. Cultural considerations in the classification of mental disorders: why and how in ICD-11. BMC Med. 2020;18:25. 31987034 10.1186/s12916-020-1493-4 PMC6983967

[15] Gureje O, Lewis-Fernandez R, Hall BJ, Reed GM. Systematic inclusion of culture-related information in ICD-11. World Psychiatry. 2019;18:357–8. 31496107 10.1002/wps.20676 PMC6732693

[16] Gaebel W, Reed GM, Jakob. Neurocognitive disorders in ICD-11: a new proposal and its outcome. World Psychiatry. 2019;18:232–3. 31059606 10.1002/wps.20634 PMC6502414

[17] McElroy E, Shevlin M, Murphy S, Roberts B, Makhashvili N, Javakhishvili J, et al. ICD-11 PTSD and complex PTSD: structural validation using network analysis. World Psychiatry. 2019;18:236–7. 31059609 10.1002/wps.20638 PMC6502420

[18] Bryant RA. Post-traumatic stress disorder: a state-of-the-art review of evidence and challenges. World Psychiatry. 2019;18:259–69. 31496089 10.1002/wps.20656 PMC6732680

[19] McCabe GA, Widiger TA. A comprehensive comparison of the ICD-11 and DSM-5 section III personality disorder models. Psychol Assess. 2020;32:72–84. 31580095 10.1037/pas0000772

[20] Bach B, Kerber A, Aluja A, Bastiaens T, Keeley JW, Claes L, et al. International assessment of DSM-5 and ICD-11 personality disorder traits: toward a common nosology in DSM-5.1. Psychopathology. 2020;5:1–10. 10.1159/000507589 32369820

[21] Zandersen M, Parnas J. Borderline personality disorder or a disorder within the schizophrenia spectrum? A psychopathological study. World Psychiatry. 2019;18:109–10. 30600641 10.1002/wps.20598 PMC6313234

[22] Stein DJ, Szatmari P, Gaebel W, Berk M, Vieta E, Maj M, et al. Mental, behavioral and neurodevelopmental disorders in the ICD-11: an international perspective on key changes and controversies. BMC Med. 2020;18:21. 31983345 10.1186/s12916-020-1495-2 PMC6983973

[23] World Health Organization. GCP.Network, https://gcp.network.

[24] Rebello TJ, Keeley JW, Kogan CS, Sharan P, Matsumoto C, Kuligyna M, et al. Anxiety and fear-related disorders in the ICD-11: results from a global case-controlled field study. Arch Med Res. 2019;50:490–501. 32018071 10.1016/j.arcmed.2019.12.012

[25] Luciano M, Sampogna G, Del Vecchio V, Giallonardo V, Palummo C, Pocai B, et al. The Italian ICD-11 field trial: clinical utility of diagnostic guidelines for schizophrenia and related disorders. Int J Ment Health Syst. 2020;14:4. 31998405 10.1186/s13033-020-0338-z PMC6979076

[26] Ng RMK. WPA educational initiatives: where are we after three years? World Psychiatry. 2020;19:257–8. 32394583 10.1002/wps.20751 PMC7214956

[27] Schulze TG. The WPA Education, Science, Publication, and Research Initiative (ESPRI): jumpstarting scientific projects in low- and middle-income countries. World Psychiatry. 2020;19:123–4. 31922685 10.1002/wps.20709 PMC6953573

[28] Giallonardo V. ICD-11 sessions within the 18th world congress of psychiatry. World Psychiatry. 2019;18:115–6. 30600609 10.1002/wps.20612 PMC6313687

[29] Perris F. ICD-11 sessions at the 19th world congress of psychiatry. World Psychiatry. 2020;19(2):263–4. 32394556 10.1002/wps.20754 PMC7214945

